# A Cognitive Autopsy Approach Towards Explaining Diagnostic Failure

**DOI:** 10.7759/cureus.17041

**Published:** 2021-08-09

**Authors:** Pat Croskerry, Sam G Campbell

**Affiliations:** 1 Division of Continuing Medical Education, Dalhousie University, Halifax, CAN; 2 Emergency Medicine, Dalhousie University, Halifax, CAN

**Keywords:** diagnostic failure, medical error

## Abstract

Diagnostic failure has emerged as one of the most significant threats to patient safety. It is important to understand the antecedents of such failures both for clinicians in practice as well is those in training. A consensus has developed in the literature that the majority of failures are due to individual or system factors or some combination of the two. A major source of variance in individual clinical performance is cognitive and affective biases; however, their role in clinical decision making has been difficult to assess partly because they are difficult to investigate experimentally. A significant drawback has been that experimental manipulations appear to confound the assessment of the context surrounding the diagnostic process itself. We conducted an exercise on selected actual cases of diagnostic errors to explore the effect of biases in the ‘real world’ emergency medicine (EM) context.

Thirty anonymized EM cases were analysed in depth through a process of root cause analysis that included an assessment of error-producing conditions (EPCs), knowledge-based errors, and how clinicians were thinking and deciding during each case. A prominent feature of the exercise was the identification of the occurrence of and interaction between specific cognitive and affective biases, through a process called cognitive autopsy. The cases covered a broad range of diagnoses across a wide variety of disciplines. A total of 24 discrete cognitive and affective biases that contributed to misdiagnosis were identified and their incidence recorded. Five to six biases were detected per case, and observed on 168 occasions across the 30 cases. Thirteen EPCs were identified. Knowledge-based errors were rare, occurring in only five definite instances. The ordinal position in which biases appeared in the diagnostic process was recorded. This experiment provides a baseline for investigating and understanding the critical role that biases play in clinical decision making as well as providing a credible explanation for why diagnoses fail.

## Introduction

Medical error is now considered a leading cause of death in the United States [1]. Of the wide variety of medical errors, diagnostic failure appears to be a significant if not the most important contributor, occurring in 10-15% of cases, and may be associated with a high degree of morbidity and mortality. It appears that physician’s clinical judgment, their cognition, how they think, bears the brunt of diagnostic failure [2]. Like all humans, physicians are vulnerable to failures in rationality, and leading cognitive scientists have identified cognitive and affective biases as the major impediment to attaining rationality [3]. Multiple types of biases in medicine were described almost two decades ago [4]. Thus, we might have better insights into clinical decision-making malfunction if we had a better understanding of the behaviour of biases in real clinical situations. With undifferentiated patient presentations, new doctor-patient interactions, high levels of uncertainty, low signal to noise ratios, and a constantly changing environment, the emergency department (ED) has been described as a ‘natural laboratory for error’, and thus an optimal clinical setting in which to study forms of cognitive failure [5].

A major difficulty in studies of bias in medicine lies in their experimental (in vitro) investigation. The diagnostic process is extraordinarily complex with upwards of about 50 factors involved [6], adding significant context to any clinical decision that is made. The classic experimental approach in which the impact of specific independent variables (e.g. characteristics of the decision maker, homeostatic challenges in the decision maker such as fatigue, cognitive loading, sleep deprivation, and context) on the dependent variable (diagnostic competence) is assessed requires the control or elimination of as many independent variables as possible. But this process inevitably isolates the diagnostic process from the very properties by which it is characterised in the clinical setting, leading to serious challenges to the external and ecological validity of this approach. As Gruppen and Frohna put it: “…too often, studies of clinical reasoning seem to take place in a vacuum. A case or scenario is presented to subjects, usually in written form, stripped of any ‘irrelevant’ noise. The traditional methodology of providing clinical cases that are decontextualized and ‘clean’ may not be a particularly valid means of assessing the full range of processes and behaviors present in clinical reasoning in natural settings [7].” Thus, the relevance of such studies towards understanding real-life clinical practice may be seriously questioned. The common method of studying diagnosis using computer screens that display clinical vignettes deprives the cases of their clinical context, resulting in a limited understanding of what actually happens in clinical practice, yet this is the dominant methodology. A review of studies of cognitive biases and heuristics in medicine found that 77% of 213 studies reviewed were based on hypothetical vignettes [8]. This de-contextualizing that occurs by studying diagnosis in the laboratory setting compromises the ecological validity of such studies. Instead, Wears and Nemeth proposed abandoning laboratory studies in favour of ‘real world’ studies that focus on context and on the intuitive processing that experts use [9]. Post hoc analyses can be done to reconstruct the clinician’s experience at the time of the event, by interviewing the clinician using cognitive interviewing techniques, and by promoting self-awareness and introspective processes [10]. Thus, the phenomenon may be studied in vivo, in the actual context in which it occurs. Reason referred to this approach as ‘corpus gathering’ - the first step in the process of classification of error [11].

The present report describes our experience of applying the approach of a cognitive autopsy to investigate the interacting contributors of various error producing conditions (EPCs), especially cognitive biases, to explore causes of diagnostic failure.

## Materials and methods

A total of 30 clinical cases were selected from a database of cases in which a diagnostic failure of significance had been identified, and which had been referred for review to the clinical chief of either of two EDs, with annual censuses of 40,000 and 75 000 visits, respectively [12]. Both clinical chiefs conducting case evaluations were well-versed in cognitive aspects of clinical decision making and were continuously involved in clinical and didactic teaching on the subject. 

To be included, each case needed to represent the standard process of evaluation in an emergency department: Patient presents to the ED → is seen and assessed by one or more physicians → may complete diagnostic imaging and laboratory investigations → is diagnosed → disposition is arranged. Any cases that did not follow this ‘typical’ format were excluded. Thus, cases in which this sequence was changed e.g., a patient had been transferred from another department, or from another hospital into the ED, or seen recently in the same ED, were excluded. Cases were also excluded if they involved missed injuries of minimal consequence such as minor fractures, lacerations, minor foreign bodies or if they involved mainly procedural errors (e.g., failed intubations, failed central lines, poor application of a cast, poor suturing technique, minor protocol violations). 

To shed light on the origins of diagnostic failure, efforts were made to document as much collateral information as possible, including prevailing conditions in the ED, and to elicit the opinions of clinicians and nursing staff about what had contributed to the outcome of the case. A key requirement for inclusion was that the emergency physician involved had to be willing to engage in an intensive process of ‘cognitive autopsy’, providing comprehensive detail about the case, and taking part in guided reflection in an attempt to understand his or her clinical reasoning and decision making through one’s involvement in the case.

We sought to identify concurrent EPCs and any evidence of cognitive or affective biases, or other notable cognitive failures including knowledge deficits, and logical failures in reasoning. Current definitions of cognitive biases in the medical setting were followed [13]. A summative assessment and evaluation of the biases, EPCs, and knowledge deficits was made to determine if any particular patterns emerged. Also, an ordinal position analysis of identified biases was completed to determine if specific biases appeared in any particular sequence in the course of a typical case.

## Results

A wide variety of clinical diagnoses was apparent, reflecting the range of conditions that can be associated with misdiagnosis, typical of any general ED. There was very little overlap in diagnoses and most disciplines were well represented. No physicians declined taking part in the process.

In all, 25 discrete biases were identified along with their respective frequencies in these 30 clinical cases (Table 1). Typically, there were five to six biases per case. Anchoring was identified as the most common bias, followed by confirmation bias, diagnosis momentum, premature closure and unpacking failure. The total number of occasions on which a discrete bias was identified was 168.

**Table 1:** Biases in the 30 cases: frequency and ordinal position

Bias	No.	Mean ordinal position
Anchoring and adjustment	16	1.69
Confirmation bias	10	5.10
Diagnosis momentum	10	4.30
Premature closure	10	3.89
Unpacking failure	10	4.70
Search satisficing	9	3.00
Affective influence	8	3.50
Ascertainment	8	1.88
Framing	8	1.87
Fundamental attribution error	8	4.63
Triage cueing	8	2.25
Psych-out error	8	4.50
Availability	7	2.14
Posterior probability error	7	3.43
Omission error	6	4.50
Representativeness	6	3.33
Commission error	5	4.60
Groupthink	5	3.80
Overconfidence	5	3.80
Authority gradient	3	3.67
Inattentional blindness	3	4.00
Belief bias	2	6.50
Gender	2	4.50
Yin-yang out	2	6.00
Zebra retreat	2	5.00

The 30 cases were analysed for other EPCs which might have predisposed clinicians and staff to error. Twelve were apparent in which an association between the EPC and an error appeared causative or at least contributory. Fatigue was the most common (Table 2). A total of 28 instances of EPCs were identified, representing potential risk factors for patient safety events (PSEs), and which appeared to contribute to adverse outcomes in some cases. Actual knowledge deficits were uncommon and were identified in only five cases (Table 3). Attending Emergency Physicians were responsible for a definite knowledge deficit in three of these cases with trainees in the other two.

**Table 2:** Error-producing conditions RACQITO, resource availability - continuous quality improvement trade-off

Rank no.	Condition	Frequency
1	Fatigue	7
2	Sleep deprivation	4
3	High-stress situation	3
4	Corridor consultation	3
5	Handover/transition of care	2
6	Time pressure	2
7	RACQITO	2
8	Cognitive overload	1
9	Rapid task switching	1
10	Poor feedback	1
11	System (technical) failure	1
12	Time delay error	1

**Table 3:** Analysis of knowledge deficits in 30 clinical cases PE, pulmonary embolus; CXR, chest X-ray; EP, emergency physician

Knowledge deficit	Comment
A resident did not appear to be aware that a normal CXR does not exclude PE.	He appeared to conclude PE was absent on the basis of the CXR
EP was not aware that protracted vomiting could result in a petechial rash of the head and neck. He attributed the rash to another cause (meningococcemia).	He acknowledged that he didn’t know of the relationship between raised intra-thoracic pressure and injury to superficial blood vessels.
The EP failed to recognise the appearance of imperforate hymen, mistaking it for an emerging fetal head	The EP said he was aware of the condition of imperforate hymen but had never seen a case of it, nor had he ever seen a photo of it.
A clinical clerk did not examine the patient’s eyes in a case of herpes zoster involving the face and nose.	He was not aware that ophthalmic involvement had to be excluded in a herpetic rash of the face.
The EP did not include a renal stone on the differential diagnosis of a patient with Crohn disease and abdominal pain..	The EP admitted that he was unaware that up to 50% of patients with Crohn disease have renal stones that likely resulted in him not putting it on the differential.

## Discussion

Although a number of studies from most disciplines in medicine have been reported illustrating the impact of particular biases, the accounts have typically been for individual biases acting in isolation, and usually without significant contextual detail [14]. An exception is a recent study in which multiple biases were studied in a series of high-fidelity clinical simulations [15]. This innovative study design avoided some of the difficulties associated with in vitro studies, but thus far these studies are rare. In the future, it may be possible to build more such in simulo

 cases using the supportive detail of cases that have been subjected to cognitive autopsy.

To our knowledge, the present study is the first to catalog the behaviour of and potential interaction between significant numbers of biases in their natural clinical setting, together with their juxtaposition to each other, alongside knowledge deficits, EPCs, and workplace conditions prevailing at the time.

Recently, Dror et al have proposed two biases that involve knock-on effects; snowball bias and cascade bias [16]. With snowball bias: ‘as one piece of evidence influences another, then greater distortive power is created because more evidence is affected (and affecting) other lines of evidence, causing bias with greater momentum, resulting in the increasing snowball of bias’, while cascade bias ‘arises as a result of irrelevant information cascading from one stage to another, e.g., from the initial evidence collection to the evaluation and interpretation of the evidence.’ In some respects, both bear similarities to triage cueing and diagnosis momentum in medicine when, for example, paramedics at the scene, or triage personnel at a later stage, form initial impressions that later turn out to be irrelevant and even misleading. Seshia et al. describe a potentiated form of bias referred to as ‘cognitive biases plus’ where several cognitive phenomena e.g., cognitive biases, conflict of interest, logical fallacies, and ethical violations, may augment and interact with each other to collectively distort clinical decision making [17].

All biases reported here were qualified as ‘probable’ as there is no tangible proof of their occurrence; they are usually invisible. However, the cognitive sciences literature is very specific about the definitions of individual biases, so there should be a reasonable correspondence between them and the behaviour to which they have been applied.

An additional issue concerns hindsight bias. Importantly, a distinction needs to be made between hindsight and hindsight bias. Hindsight is learning from experience, an essential part of human behaviour, whereas hindsight bias is usually a subconscious tendency to distort the past to make the decision maker appear more or less favourably than they actually were. The benefit of true hindsight is that if relevant information is objectively and knowledgeably assessed, bias may be minimal, and important insights may be gained.

Many physicians have had little experience in purposeful introspection, reflection, insight into, or knowledge of the cognitive failures described in the majority of cases reported here. Typically, when cases of diagnostic failure come to light clinicians often make self-recriminatory judgements and comments such as “Well, I guess I dropped the ball there”, or “I screwed that one up”, rather than analysing them in any detail or seeing their potential as opportunities for learning or for adjusting cognitive habits. Mostly, this reluctance appears to be due to a lack of awareness of the nature and extent of cognitive biases and how they impact clinical decision making. This may not be surprising in view of the lack of training in these areas in many current medical curricula. Multiple obstacles to understanding diagnostic failure have been described [18] which, along with the sheer complexity of the process [6] provide some explanation for why diagnostic failure has been under-estimated in the past and has taken so long to move into the spotlight in patient safety. In the present study, we found that physicians appreciated the process and were more self-forgiving after having their unconscious biases revealed to them in retrospect, recognizing them as relatively predictable human foibles.

It has been argued that as errors arising in intuitive (System one) decision making are due to unconscious processes, they are not available to introspection [19]. While it is true that most System 1 processes are autonomous and outside of conscious control at the time they are triggered, an awareness that they have occurred may develop and be made consciously available through introspection, mindfulness and reflection, processes by which cognitive bias mitigation (CBM) can occur [20-21]. In order to improve our understanding of the diagnostic process, more attention needs to be directed towards its multiple facets, context, and overall complexity [6]. An important observation from the present cases is that biases rarely occur in isolation. It seems likely that once certain biases occur others inevitably follow.

The psychology literature is very clear about the abundance of biases in decision making and the present experiment provides further support. Although medical students and other novitiates to the domain of cognitive biases may despair at the sheer number of them, currently estimated in the order of about 200 [22], it appears that the actual number that occur commonly may be quite limited. If medical trainees could at least identify these and have some understanding of how they work, it would be a significant step towards attaining awareness and engaging strategies to mitigate their action [21]. This is critically important for developing expertise in clinical decision making.

**Figure 1:**
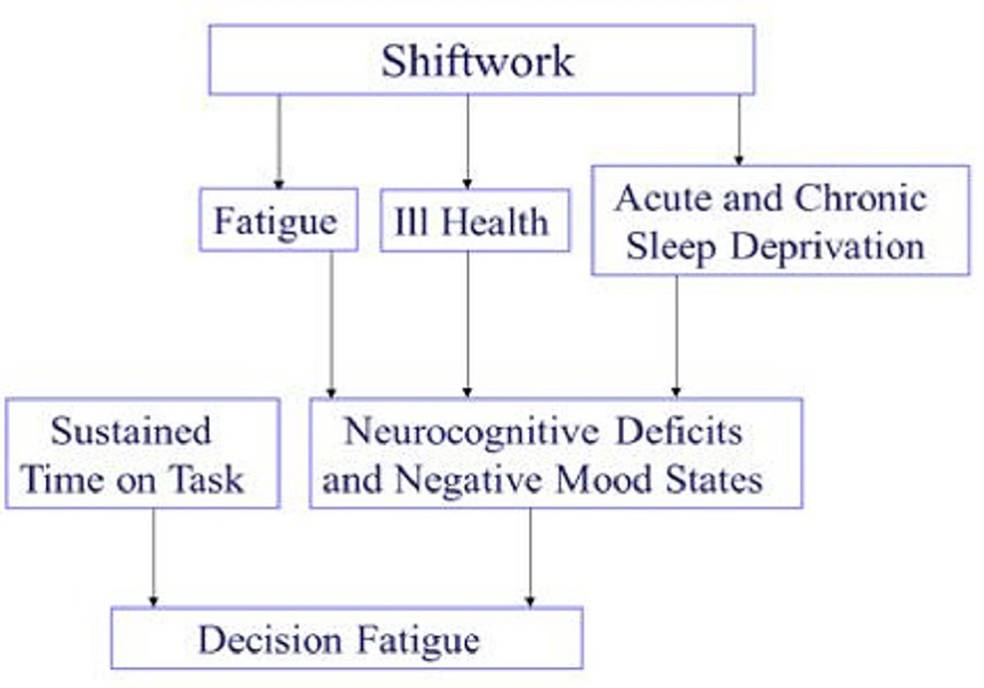
Contributory factors in decision fatigue

Although a number of instances of EPCs were identified in the present series it is the author’s experience from this and several other EDs that deviance from accepted standards of patient safety is normalised in many departments to the point that they hardly attract attention or comment. Fatigue, stress, intermittent cognitive loading, extended lengths of stay in the ED, interruptions and distractions, rapid task switching and other EPCs have become commonplace and escape notice much of the time. Thus, in ED studies we might expect them to be under-reported. Fatigue was the most common EPC observed here (Table 2). Even when workers might not be subjectively experiencing fatigue, there is mounting evidence that decision fatigue begins to set in after several hours of sustained work [23]. Decision fatigue has been attributed to a decline in executive function, localized in the prefrontal cortex of the brain, likely associated with an increased use of heuristics, and a decline in quality of decision making [24]. In some of the cases reviewed here, fatigue was very evident, but in others decision fatigue may have influenced outcome in more subtle ways. Further, sleep deprivation and sleep debt which are common in emergency medicine, are inevitably associated with fatigue in several ways (Figure 1). Handover represents a transition of care from one clinician to another and is known to be a vulnerable point in patient care [25]. Resource Availability Continuous Quality Improvement Trade-Off (RACQITO) is based on the well-known SATO (speed accuracy trade- off) described in the psychology literature. As resources become increasingly limited the quality and safety of care may become more compromised. Notably, system failures were very rare; the only instance was in a case where the radiology technician was unable to transmit images to the ED, which led to delay and suboptimal viewing of the images in another location.

The incidence of knowledge deficits in this study was low. We found unequivocal knowledge deficits in only three instances by the attending emergency physician (Table 3). This is consistent with that found in a previous study where they were estimated at about 3% [2], and confirming the observations of Kieswetter et. al [26]. Commonly, when clinicians realise they have made a mistake and have got past the initial defence mechanisms of denial, distancing, and discounting, a typical self-recriminatory response is to feel that they don’t know enough. Similarly, when a diagnosis has failed, some patients believe that their doctors did not know enough. Other studies have claimed explicitly that a principal cause of diagnostic failure is a knowledge deficit on the part of the decision maker, with the authors of one study indicating that several knowledge-related factors co-occur, with physicians either not possessing sufficient knowledge or not applying their knowledge correctly [27]. Although these two possibilities were considered together, there is an important difference between them. It is one thing to misdiagnose a patient due to lack of knowledge about their disease, but quite another to misdiagnose them because the clinician simply did not consider a diagnosis despite knowing its clinic-pathological features in detail. The present study shows that across a wide range of significant misdiagnoses, knowledge deficits were relatively rare, and an uncommon cause of diagnostic failure.

In contrast to the paucity of knowledge errors, cognitive biases were abundant and appeared significantly more consequential. Our study suggests that failing to apply medical knowledge (how to think) because of cognitive biases is far more common and more consequential than a simple lack of medical knowledge (what to think). Thus, a distinction needs to be made between medical knowledge that embraces the traditional content of a medical curriculum which covers a wide range of facts about anatomy, physiology, pathophysiology, and the management of disease, versus an expanded domain that included knowledge of cognitive science, in particular the influence of heuristics and cognitive biases on human decision making. Because medical curricula have traditionally not covered these developments in cognitive science, medical graduates would be expected to have knowledge deficits in these areas. These deficits are distinguished from individual personal qualities such as carelessness, arrogance, gullibility, naivety, incuriousness, wishful thinking, unwariness and other character traits and attitudes: i.e. things that may get in the way of knowledge at a subconscious level. This is also an important distinction as the common usage and understanding of ‘bias’ inclines more towards its negative aspects i.e., as a vice (and therefore usually more blameworthy) rather than the unconscious, involuntary response that it typically is. It might prove easier to educate clinicians about biases if they were not seen in negative terms.

There is accumulating evidence that cognitive and affective biases are an integral part of clinical decision making, and that the same biases would be expected to impact decision-making at all levels of the healthcare system [14,17, 28]. Inevitably, patient safety is potentially threatened whenever decision making takes place. Recent consensus statements have firmly identified that recognising the limits and biases of human cognition is a foundational concept to improve diagnostic quality and safety, as is the use of reflection, surveillance, and critical thinking to mitigate their detrimental effects throughout the clinical encounter [29].

The strategy proposed here for studying clinical decision making, the cognitive autopsy, goes beyond the parameters of conventional root cause analysis. It is important for those conducting a cognitive autopsy to have a solid grounding in the cognitive science that underpins clinical decision making, with especially a detailed knowledge of the common cognitive biases, as well as an understanding of the physiological processes that underlie fatigue, stress, sleep deprivation and cognitive loading in clinical decision making.

The Oxford physicist David Deutsch, has argued that explanation is the bedrock of reason and that the overwhelming majority of theories are rejected because they contain bad explanations, not because they fail experimental verification [30]. For the diagnostic process, unless we provide a good explanation for why it fails, it is unlikely that progress will be made. To date, the failure has been variously attributed to a variety of factors: physician knowledge deficits, personal qualities of the physician, systemic factors in the healthcare environment, and, perhaps, a tacit acceptance of ‘the cost of doing business’ with a complex process. However, thus far, these factors have not provided an adequate explanation. Instead, it seems there is growing evidence and acceptance that diagnostic failure depends on how physicians think, more so than any other factor. There remains now an ethical imperative to apply the protean findings from the cognitive science literature. They provide a robust and comprehensive explanation of how the diagnostic process works.

## Conclusions

The present study has focused on cases involving diagnostic failure that were collected for review in the context of patient safety. While the causes of diagnostic failure are well-known such as system failures, deficits in knowledge, ambient working conditions, the calibration of physicians thinking, and possibly other unspecified conditions, we have not previously developed a sense of how to investigate the relative proportions of, or interaction between each. The primary objective of the present study was to use a cognitive autopsy approach to delineate those causes with a view towards a better explanation and future prevention.

With a working knowledge of cognitive and affective biases, we were able to conduct a cognitive root cause analysis of real clinical cases, at the same time recording error producing and ambient conditions, and possible knowledge deficits, in the outcomes. Despite literature on the relative paucity of knowledge-based errors, we were still surprised to find so few, testimony to the general efficacy of medical training as well as the clinical effort of practitioners. Equally surprising was the finding of so many cognitive and affective biases. What is needed now are explicit efforts to integrate cognitive science into medical curricula, such that it becomes part of core medical knowledge in graduating physicians.
