# Correction: A Cognitive Autopsy Approach Towards Explaining Diagnostic Failure

**DOI:** 10.7759/cureus.c48

**Published:** 2021-09-13

**Authors:** Pat Croskerry, Sam G Campbell

**Affiliations:** 1 Division of Continuing Medical Education, Dalhousie University, Halifax, CAN; 2 Emergency Medicine, Dalhousie University, Halifax, CAN

The present, corrected article is a revised version of one that was published several weeks ago. The first version contained a reference list from an earlier draft that was mistakenly attached. We apologize for any confusion and inconvenience that may have resulted from this error.

- Pat Croskerry and Sam Campbell

